# The Complexities of Periorbital Neurofibroma: Diagnostic Ambiguity and Therapeutic Dilemmas: A Case Report and Literature Review

**DOI:** 10.3390/diagnostics16050732

**Published:** 2026-03-01

**Authors:** Marijus Leketas, Gerda Kilinskaitė, Nida Kilinskaitė, Goda Miniauskienė, Žygimantas Petronis, Audra Janovskienė

**Affiliations:** 1 Department of Maxillofacial Surgery, Lithuanian University of Health Sciences, Mickeviciaus g. 9, LT-50103 Kaunas, Lithuania; marijus.leketas@lsmu.lt (M.L.); gerda.kilinskaite@stud.lsmu.lt (G.K.); zygimantas.petronis@lsmu.lt (Ž.P.); 2 Odontology Faculty, Lithuanian University of Health Sciences, Mickeviciaus g. 9, LT-50103 Kaunas, Lithuania; nida.kilinskaite@stud.lsmu.lt; 3 Department of Ophthalmology, Lithuanian University of Health Sciences, Mickeviciaus g. 9, LT-50103 Kaunas, Lithuania; goda.miniauskaite@lsmu.lt

**Keywords:** periorbital tumors, neurofibroma, NF1, melanoma

## Abstract

**Background:** Periorbital tumors represent a diagnostic challenge due to overlapping clinical and histopathological features. **Case presentation:** We present the case of a 57-year-old female with a recurrent left lower eyelid lesion initially diagnosed as malignant melanoma. Over a seven-year course, the patient underwent multiple surgical excisions, radiotherapy, systemic therapies, and repeated imaging. Histopathological findings alternated between melanoma, neuroma, hybrid peripheral nerve sheath tumor, and ultimately neurofibroma (NF1). Immunohistochemical staining repeatedly demonstrated positivity for S100 and SOX10, with variable expression of melanocytic markers, underscoring the diagnostic ambiguity between desmoplastic melanoma and NF. Despite multiple interventions, including Pembrolizumab therapy and orbital exenteration, tumor progression persisted. This case highlights the considerable difficulty in distinguishing melanoma from neurofibroma in the periorbital region, particularly when histological and immunohistochemical profiles overlap. **Conclusions:** Accurate diagnosis requires a multidisciplinary approach, repeated reassessment, and awareness of rare presentations. Our report emphasizes the importance of integrating clinicopathological data and selected molecular diagnostics to optimize management of such complex cases.

## 1. Introduction

Melanoma, a formidable entity in dermatology, exhibits diverse manifestations, ranging from primary orbital disease to secondary involvement due to regional extension or metastasis. Globally, the incidence of melanoma has been steadily increasing over the past decades, particularly in fair-skinned populations. Cutaneous melanoma accounts for the vast majority of cases, with an estimated annual incidence exceeding 300,000 new cases worldwide. In contrast, Uveal Melanoma represents the most common primary intraocular malignancy in adults but remains relatively rare, with an incidence of approximately 5–7 cases per million population per year. Risk factors include ultraviolet radiation exposure, genetic predisposition, fair skin phenotype, and preexisting melanocytic lesions. Despite advances in targeted and immunotherapy, melanoma continues to be responsible for significant morbidity and mortality, particularly in advanced stages [1,2]. Clinical presentations often include diplopia, ptosis, abnormal eye movements, chemosis, and conjunctival injection, with advanced cases demonstrating optic nerve compression and observable optic disc swelling [3,4,5].

The primary occurrence of periorbital melanoma is thought to arise from the leptomeninges, ciliary nerves, or ectopic nests of melanocytes within the orbit. Diagnosis is confirmed using specific immunohistochemical markers, such as S100, HMB-45, and Melan-A. S100 demonstrates high sensitivity, with positive expression reported in approximately 95–100% of melanomas, whereas HMB-45 and Melan-A show positivity in approximately 70–90% and 75–92% of cases, respectively [6]. Surgical intervention remains the cornerstone of treatment, including local resection, debulking, or exenteration, often complemented by adjuvant radiotherapy [7]. Despite these therapeutic efforts, prognosis remains a concern, with a median overall survival of 36.9 months and a 5-year survival rate of 42% [8].

In parallel, neurofibromas (NFs), although relatively uncommon in the periorbital region, constitute approximately 2–4% of periorbital tumors [8]. They are characterized by progressive symptoms such as proptosis, globe displacement, impaired extraocular motility, ptosis, sensory deficits, and occasionally optic neuropathy. NFs arise from peripheral nerve sheath tumors with diverse subtypes [9]. These lesions, composed of Schwann cells, axons, perineural cells, and fibroblasts, often embedded in a collagenous and myxoid matrix, may show S100 positivity on immunohistochemical studies. S100 expression is typically observed in the majority of cases, with reported positivity rates ranging from 80 to 100%, reflecting the Schwann cell origin of these tumors [9,10]. Localized NFs present with varying clinical severity, prompting management strategies ranging from observation to surgical excision via anterior or lateral orbitotomy [11]. The risk of malignant transformation, particularly in Neurofibromatosis type 1 (NF1), requires vigilant follow-up, as up to 20% of cases may progress to malignancy [12].

This report highlights the complexities of an extraordinary case in which the diagnostic landscape was obscured, necessitating a nuanced approach to tumor management and surgical intervention. The convergence of primary periorbital melanoma and NF in a single patient makes this case unique because it illustrates diagnostic ambiguity, long-term follow-up, and immunohistochemical challenges, providing valuable insights for clinicians and pathologists managing such rare and intricate presentations.

## 2. Literature Review

### 2.1. Epidemiology

Ocular and periorbital melanomas are the second most prevalent type of melanoma in Western countries, following cutaneous melanoma. They distinguish themselves from cutaneous melanoma through differences in molecular drivers, metastatic patterns, and the tumor-immune microenvironment [13].

### 2.2. Melanoma Treatment

Treatment for localized ocular and periorbital melanomas emphasizes complete surgical resection or radiation. However, approximately half of patients with Uveal Melanoma (UM) experience recurrence or metastasis after initial therapy, with the liver being the most common site of metastasis. Systemic therapy for metastatic ocular and periorbital melanomas shows limited efficacy without a standardized approach, with chemotherapy and immunotherapy demonstrating poor effectiveness. A recent development is Tebentafusp, an FDA-approved T-cell-redirecting bispecific fusion protein, designed for treating previously untreated HLA-A*02:01-positive patients with metastatic UM [13,14,15]. In a study by Liu X et al. [16], 51 patients with metastatic ocular and periorbital melanoma were observed. The uvea was identified as the most common primary site (73%), followed by the conjunctiva (22%), lacrimal sac (4%), and orbit (2%). Notably, patients with Uveal Melanoma (UM) exhibited distinctive characteristics compared to those with conjunctival melanoma (CM), including age, incidence of liver metastases, incidence of lymph nodes metastases, and the presence of BRAF mutation. The overall response rate to first-line treatment was 18%, with specific positive responses observed in patients with BRAF-mutated CM. The study provided insights into the median progression-free survival (PFS) and overall survival (OS) of first-line treatment, revealing correlations between liver-directed treatment and improved patient PFS and OS among those with liver metastases [16].

### 2.3. Differentiation

Throughout history, melanoma has been known for its ability to mimic various lesions, leading dermatopathologists to rely on immunohistochemical markers for diagnosis [17,18]. However, this conventional understanding does not apply to a specific variant known as “Dedifferentiated Melanoma” (DM), characterized by the loss of typical melanocytic markers [19]. DMs pose challenges for clinical and microscopic diagnosis due to their hypo- or amelanotic nature. Distinguishing DMs from lesions like scars or NFs is particularly challenging. Few studies have addressed this diagnostic challenge, and there is limited information about the natural course of neurofibroma-like desmoplastic melanomas (NFLDMs) [19,20]. Gerami P et al. [20] presents 12 cases of NFLDMs, with clinical follow-up for 8 cases. Among them, four cases experienced clinical recurrence, and two transformed into a more aggressive phenotype. The study examined PRAME, P53, and neurofilament to assess their potential in distinguishing NFs from DMs. DM differs from desmoplastic melanoma: DM often loses melanocytic markers (S100, SOX-10, Melan-A), while desmoplastic melanoma retains S100 and SOX-10. These differences, along with PRAME and p53 staining, help distinguish the two entities [20,21].

### 2.4. Main Types of NF—Diagnostic Criteria

NF is a heterogeneous group of hereditary cancer syndromes that lead to tumors of the central and peripheral nervous systems, as well as other organ systems. NF1 is by far the most prevalent type, accounting for 96% of cases followed by NF2 (3%), and a more recently recognized, lesser-known form—schwannomatosis [22].

The main types of NF are distinguished by specific diagnostic criteria, which have been revised to incorporate recent clinical and genetic discoveries. The revised criteria were developed through an international, multispecialty effort, utilizing a modified Delphi approach to balance high sensitivity and specificity for accurate diagnosis. Key criteria include pigmentary findings such as café-au-lait macules (CALMs) and skinfold freckling, which should be present bilaterally to reduce misdiagnosis of mosaic NF1. However, pigmentary findings alone are not specific for NF1, necessitating consideration of alternative diagnoses like Legius syndrome (LGSS), Noonan syndrome with multiple lentigines, and constitutional mismatch repair deficiency syndrome (CMMRD). Genetic testing and the presence of Lisch nodules of iris or choroidal anomalies can confirm NF1, especially in older individuals or those with extensive cutaneous or spinal NF. The revised criteria aim to improve diagnostic accuracy across different age groups and clinical presentations [23].

### 2.5. NF Treatment

The *NF1* gene, found on chromosome 17q11.2, encodes neurofibromin, a tumor suppressor protein that inhibits the Ras/MAPK and PI3K/mTOR signaling pathways. On chromosome 22q12, the *NF2* gene is found. It encodes merlin, a tumor suppressor protein connected to ezrin, radiatin, and moesin that modifies the activity of the mTOR, Raf/MEK/ERK, and PI3K/AKT signaling pathways. On the other hand, schwannomatosis is caused by a mutation in the SMARCB1 gene, also known as the INI1, BAF47, or hSNF5 gene, located on chromosome 22q11.2, centromeric to the *NF2* gene. Different *NF* types require different treatment strategies [24].

For the majority of NF1-related oncological problems, resective surgery is the initial treatment choice. However, local expansion and invasion of critical areas, tumor size, and danger of postoperative regrowth sometimes make for unsatisfactory outcomes. However, the high toxicity rates in *NF1* patients limit the use of radiation and chemotherapy. Additionally, only very carefully chosen patients should receive chemotherapy and radiation therapy when no alternative course of treatment is feasible [25].

In individuals with *NF2*, radiation therapy should not be the primary line of treatment for benign tumors, especially in consideration of the potential for future pharmacological interventions following the established advantages of bevacizumab [26]. Most *NF2* patients who received vestibular schwannoma (VS) treatment with proton radiation therapy (PRT) did not need further therapy. The treatment-related toxicity was substantial, much like with photon radiotherapy (PhRT). However, a comparison of the current PRT results with the PhRT literature is incorrect since, in the majority of patients in the current cohort, PRT was used as a salvage treatment following surgery and/or bevacizumab therapy. To thoroughly assess PRT’s potential benefit, a longer follow-up of prospective NF2 cohorts receiving it as the main treatment for VS is required [27]. Previous studies have confirmed that bevacizumab can control the progression of VS and symptoms associated with VS growth [28].

Neuropathic medications such as gabapentin, amitriptyline, and pregabalin are used in the traditional conservative treatment of chronic neuropathic pain linked to schwannomatosis. Surgical resection of the offending lesion is still an effective, albeit more intrusive, treatment option for patients with schwannomas producing functional restrictions, as well as for those with refractory pain and/or medication resistance [29].

There are only a few clinical cases that describe NF in the ocular area in the last 5 years. Both cases were identified as NF1 [30,31].

## 3. Case Report

A 57-year-old female reported at the Department of Ophthalmology, Maxillofacial Surgery of the Hospital of Lithuanian University of Health Sciences Kaunas Clinics. The patient reported the onset of a non-healing nodule on the lower eyelid of the left eye approximately 9 months ago. The nodule intermittently displays erythematous discoloration and occasional crusting. The patient is diagnosed with arterial hypertension and uses prescribed Nebivolol and Perindopril medications for disease’s management.

Malignant melanoma was diagnosed in April 2017 after biopsy was performed (Table 1). During the 7-year clinical follow-up period, the patient underwent nine surgeries and received one round of radiotherapy. Hospital databases contain eight biopsy results and six MRI scans for reference.

**In June 2017**, surgery was performed by excising the right inferior lower eyelid tumor and plasticizing the eyelid defect with a cartilage flap from the upper eyelid with blood supply and a flap of skin from the upper lid. Pathohistological examination revealed a malignant skin melanoma, pT2a (Table 1). MRI was used to plan the procedure. The MRI information is out of date; therefore, it is inaccessible.

For differential diagnosis, blood samples were taken to rule out NF, and the results were negative.

**In February 2019,** the patient arrived again due to swelling and hardening in the lateral third of the left lower eyelid.

**In June 2019,** left lower eyelid tumor removal and skin and cartilage plastic surgery were performed. The biopsy described that most of the data was for traumatic neuroma (Table 1).

**In November 2020,** repeated excision of the neuroma in the lower eyelid and plasty of the eyelid defect with the ear cartilage were performed.

**In September 2021**, a repeat excision of the neuroma in the lower eyelid and a plasty of the eyelid defect with the periosteum, amniotic membrane, and muscle fascia were performed.

**In March 2022,** a repeat neuroma in the lower eyelid (outer third) was excised, and plastic surgery was performed on the eyelid defect with the periosteum from the edge of the eye socket and oral mucosa. A biopsy was also performed (Table 1).

**In August 2022**, the left lower eyelid was thickened, and redness was observed on the conjunctival side. MRI was also performed (Table 2).

**In September 2022**, a tumor in the inner corner of the eyelid that spread to the orbit was removed, and skin plastic surgery was performed. The diagnosis at biopsy was a benign hybrid tumor of the nerve sheaths (Table 1).

**In October 2022,** the skin of the lower eyelid thickened and reddened again. The performed MRI revealed left extraconal lateral, preseptal, and post-septal formations that accumulated intense contrast material, possibly infiltrating the lower surface of the lacrimal gland. The renewed suspicion of melanoma was prompted by these MRI findings (Table 2).

**In February 2023,** repeated excision of the neuroma in the lower eyelid and plasty of the eyelid defect with the ear cartilage were performed. A biopsy was taken (Table 1).

**In April 2023,** MRI was performed (Table 2). The patient underwent an operation, the tumor was removed, and plastic surgery was performed with local tissues. Microscopic examination revealed that the majority of cells were positive for SOX10 immunolabeling. The proliferation index Ki67 reached 5%. Benign changes were detected, possibly indicative of NF (Table 1).

**In July 2023**, the patient noticed a new lesion in the lower eyelid of the left eye. Tumor growth was observed extraorally on the left edge of the left eye socket. Suspicious recurrence was observed on the basis of edematous changes after MRI was performed on the postoperative lodge. At the multidisciplinary conference focusing on head and neck oncological conditions, the treatment option of radiation therapy was proposed.

Radiation therapy was delivered to the region adjacent to the tumor in the vicinity of the left eye, with a dosage of 25 Gray over 5 fractions.

After unsuccessful radiotherapy, a previous biopsy was delivered to a different laboratory for a more detailed examination. An examination revealed a new diagnosis—NF1.

**In April 2024,** visually and clinically—the formation in the eye area is not growing; the condition is not worsening; visual disturbances, pain, or other symptoms are not progressing. The disease is considered stable. Therefore, treatment with Imatinib is continued.

**In June 2024,** disease progression is observed on the background of Imatinib. After assessing the biological course of the tumor, the tumor is considered malignant, the diagnosis is corrected. During the multidisciplinary consultation, it is decided to prescribe treatment with Pembrolizumab.

**Figure 1:**
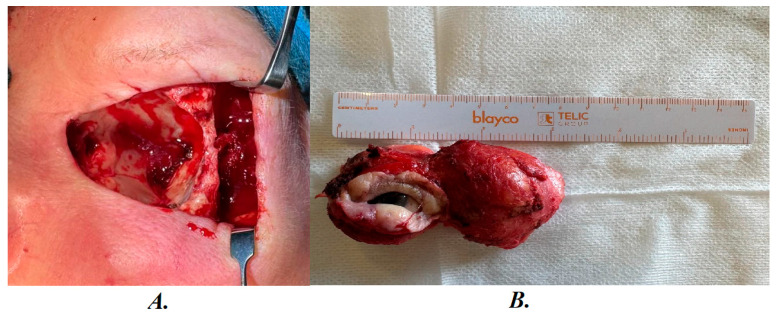
(**A**) Intraoperative view during left eye enucleation performed in October 2024, demonstrating a large intraocular mass occupying the globe. (**B**) View of the enucleated specimen measuring approximately 4–5 cm in greatest dimension. The globe was enlarged and replaced by a reddish-brown solid tumor with heterogeneous cut surface and focal areas of hemorrhage.

**In October 2024,** since the growing formations on the forehead and in the eye cause significant discomfort, it was decided to perform surgical excision of the tumor. After preparing a sterile operating field: under general anesthesia, an incision was made at the upper and lower orbital rims, the soft tissues of the orbit with tumor masses were bluntly separated from the soft tissues, the optic nerve and blood vessels were accessed, cauterized, the soft tissues of the left orbit with tumor masses were removed in one conglomerate, and submitted for examination (Figure 1). Tissues from the forehead and left cheek area were released, the submucosal layer was sutured, and a sterile bandage was applied. NF was detected during the biopsy (Table 1).

Histopathological examination revealed a moderately cellular tumor overgrowing the globe, extending to the eyelids, and infiltrating the adjacent muscle tissue, with focal involvement of the surgical margins. The tumor was composed of spindle-shaped and polygonal cells arranged partly in a storiform pattern, with oval to elongated nuclei and moderate cytoplasm. Mitotic figures were rare. Moderate mononuclear inflammatory infiltration was present in the stroma. Immunohistochemically, tumor cells were positive for S100 and p16, and negative for EMA, calretinin, and CD34. The Ki-67 proliferation index was low (5–10%).

Final histopathological diagnosis: benign peripheral nerve sheath tumor, most consistent with neurofibroma (NF).

### Magnetic Resonance Imaging

**Figure 2:**
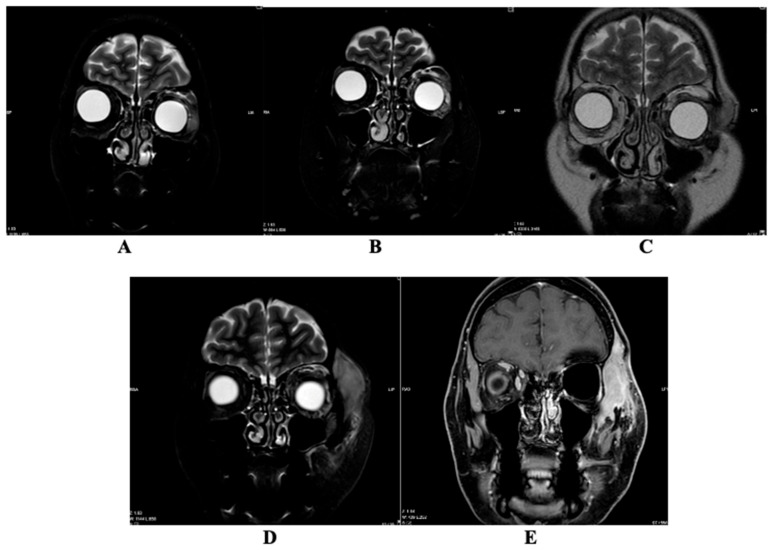
Magnetic resonance imaging (MRI) changes on a coronal plane showing lesion-related changes in the left orbital region. (**A**) MRI performed 21 December 2022; (**B**) MRI performed 7 June 2023; (**C**) MRI performed 27 May 2024; (**D**) MRI performed 14 August 2024; (**E**) MRI performed 8 January 2025.

**Figure 3:**
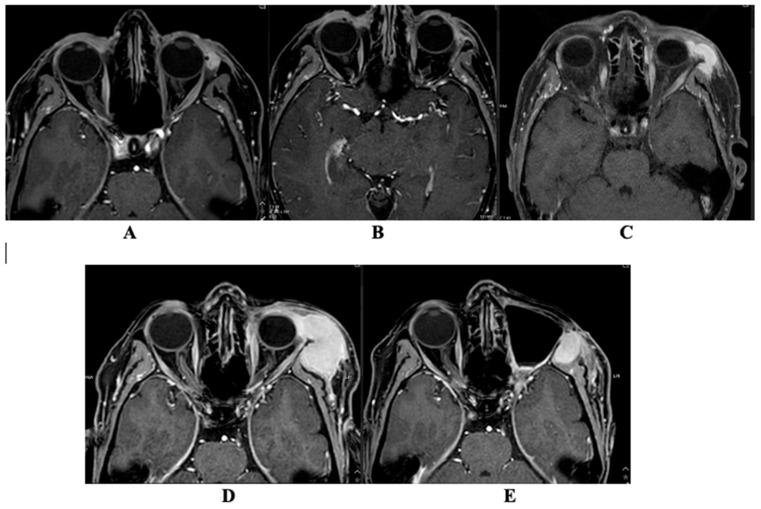
Magnetic resonance imaging (MRI) changes on an axial plane showing lesion-related changes in the left orbital region. (**A**) MRI performed 21 December 2022; (**B**) MRI performed 7 June 2023; (**C**) MRI performed 27 May 2024; (**D**) MRI performed 14 August 2024; (**E**) MRI performed 8 January 2025.

Six magnetic resonance imaging (MRI) scans have been archived in the hospital databases within 6 years (Table 2). Unfortunately, the images from the first MRI performed in 2022 are no longer available for display; only the description is retained. The remaining five MRI scans are shown in Figure 2 and Figure 3.

MRI was performed using T1-weighted spin echo (T1W_SE), 3D T1-weighted turbo field echo (3D TFE), STIR (long TE), T2-weighted turbo spin echo (T2W_TSE), 3D T1-weighted mDIXON sequences pre- and post-contrast, as well as DWI/ADC imaging. Axial, coronal, and sagittal planes were acquired. Intravenous contrast enhancement was performed using 13 mL of gadoterate meglumine (Clariscan^®^, GE HealthCare, Chicago, IL, USA).

In the MRI conducted on 17 August 2022, a well-defined gray signal intensity lesion measuring 9.3 × 17.7 × 10.2 mm was observed medially extraconally on the left side, extending from the eyelid to the middle portion of the eyeball. This lesion exhibited intense contrast agent enhancement. After the surgical procedure, on 21 December 2022, a further MRI examination was performed, during which there was a 0.9 × 1.8 × 2.8 cm reduced mass on the left side, accumulating contrast material, with clear edges, located laterally extraconically in the lower orbit. In the MRI study performed on 7 June 2023, at the height of the lower eyelid of the left eye and in the postoperative area, pre-/post-septal tissue was uneven MR SI due to postoperative-edematous changes; against their background, in the medial area of the lower eyelid, a ~17 × 7 × 9 mm mildly hyperintense for muscles, intensive contrast agent accumulating formation, similar to the formation seen on 21 December 2022 on MRI. At the time of this MRI examination, there was a suspected recurrence of the disease. In the MRI scan performed on 27 May 2024, the lesion in the lower eyelid of the left eye and the postoperative area in the medial-middle region of the lower eyelid increased in size to 26 × 30 × 14 mm. This MRI revealed multifocal infiltration progression of the tumor within the left intra-extraorbital region. In the MRI scan performed on 14 August 2024, in the lower eyelid area of the left eye and in the postoperative bed pre-/postseptally medial—middle lower eyelid area, the formation has slightly decreased to ~26 × 28 × 13 mm. In the lateral middle preseptal part of the orbit and above, intra-extraorbitally superolaterally localized formations merged into one formation that significantly increased in dynamics to ~29.5 × 43.4 × 42.6 mm in size. This MRI showed progression of the oncological process. MRT was performed on 8 January 2025 after repeated non-radical tumor removal surgery with orbital exenteration. In the left temporal fossa—the volume of the perizygomatic tumor tissue has decreased to ~1.9 × 1.7 × 4.1 cm and the pathological node in the anterior part of the superficial lobe of the left parotid salivary gland preauricularly has significantly decreased to ~0.6 × 0.4 cm in size coronally.

## 4. Discussion

Melanoma and NF pose a diagnostic challenge due to their overlapping clinical and histopathological features. Both conditions can manifest as pigmented skin lesions, making visual inspection alone insufficient for accurate differentiation. Additionally, the presence of melanocytes in NF and the potential pigmentation of these lesions further complicate the distinction between the two entities.

It can be especially challenging to differentiate between desmoplastic melanomas (DMs) and NF. With DM, from a histopathological perspective, without clear signs of melanoma in situ, the fibrous dermal spindle cell growth may be misinterpreted as a scar, dermatofibroma, or NF, particularly during a superficial shave biopsy [32]. Nevertheless, while immunohistochemical (IHC) studies can effectively differentiate a fibrosing melanocytic neoplasm from a scar or dermatofibroma with the right biopsy, distinguishing it from a NF is more complex. This is particularly true because certain forms of DM may exhibit characteristics of “neural transformation” or Schwannian differentiation that closely resemble or are, in some areas, indistinguishable from a NF [33].

In terms of the clinical follow-up of our patient spanning a period of 7 years, the patient underwent a total of nine surgical procedures and received one course of radiotherapy. There were seven biopsy findings made and four MRI scans accessible within the hospital databases.

A diagnosis of malignant melanoma was made at the initial biopsy. In initial biopsy, tumor cells were positive for *S100P* and *Melan-A* immunolabels; some cells reacted with *HMB45* immunolabel. Tumor cells did not react with *CK5*, *CD1a*, and *CD68* immunolabels. This was followed by surgical excision of the tumor, which again revealed a malignant, possibly DM. During biopsy examination, tumor cells reacted with the *S100P* marker again. This time *Melan-A* did not react with smooth muscle actin. *CD34*, *EMA*, *CD56*, *Synapt*, and *CD68*, *Ki 67*—proliferative index was high in the junction area, lower in the dermis. In 2020, after a traumatic neuroma was detected during an excisional biopsy, spindle-shaped cells and small nerve fibers was positive for *S100P* immunolabel. The reaction with total *CK*, *HMB45*, and *Melan-A* markers was negative. A biopsy in 2022 diagnosed a neuroma in which the majority of tumor cells reacted positively with immunolabels *S100P* and *SOX10*. April 2023 in a biopsy that diagnosed a neuroma described spindle-shaped cells positivity for *S100P*, *SOX10* immunolabels. After being diagnosed with NF in 2023 July, the majority of cells were positive for the *SOX10* immunolabel [Table 1].

The issue at hand pertains to the fact that the immunophenotype for both entities is essentially identical, as both DM and NF contain S100P positive spindled cells, express SOX-10, and generally lack expression of Melan-A and HMB-45—although in the first biopsy Melan-A was positive, which may have prompted the initial melanoma diagnosis; after subsequent surgical excision evaluations, Melan-A was negative, highlighting the diagnostic challenge [34,35,36,37,38].

The favorable aspect is that CD34 presents a promising prospect as a discriminative marker distinguishing DM from NF. CD34 represents an immunoreagent with broad reactivity that recognizes a transmembrane sialoprotein, expression of which can occur in cells of mesenchymal, hematopoietic, and follicular outer sheath lineage [39]. In the context of neural and melanocytic proliferations, sometimes such a configuration evokes a differential diagnosis of junctional melanocytic nevus combined with NF vs. early DM [21]. Yeh I et al.’s [21] study notices that a lack of CD34 expression is in association with DM. Immunochemistry was performed and CD34 labeling was present in greater than 30% of the proliferation in 96% of NF and in only 4% of DM. Over two-thirds of the NF exhibited a CD34 fingerprint involving more than 60% of their surface area. In the two cases of DM that showed CD34 fingerprint positivity, the staining was patchy and involved less than 60% of the tumor. In partially staining NF, areas without a CD34 fingerprint tended to occur in central lobular areas. This study revealed that CD34 fingerprint immunoreactivity could be useful in distinguishing NF from early DM—in our case, CD34 was also positive after the first surgical excision in 2017 [21].

Gerami P et al.’s [20] study analyzed PRAME, P53, and neurofilament staining possibility to serve as a diagnostic approach to distinguish between DM and NF. However, these immunohistochemical evaluations (PRAME and P53) were not performed in our patient. In all NFS instances, there was some localized positive staining observed with neurofilaments of axonal components, a characteristic also present in the majority of DM cases. Therefore, immunohistochemical examination with neurofilament seems to be not an accurate diagnostic method at this time. Some level of PRAME positivity was seen in 8 of 10 cases and P53 positivity in 6 of 10 cases of DM. Therefore, the detection of PRAME and P53 positivity, along with either a total absence of staining or a significant cluster of tumor cells showing positive staining, would lean towards a diagnosis of DM rather than NF. These results align with previous studies that have examined the prevalence of positive P53 and PRAME staining in DM [40,41].

Furthermore, given the potential associations between DM and NF, individuals with NFS type 1 (NF1) have an increased risk of developing melanoma, adding to the complexity of distinguishing between these conditions. Rubinstein TJ et al.’s [42] study presents a case in which a 56-year-old woman with NFS type 1 (NF1) presented with a left upper eyelid amelanotic nodule with adjacent eyelid margin hyperpigmentation. Physical examination additionally revealed primary acquired melanosis (PAM) on the palpebral conjunctiva of the same eyelid. Full thickness eyelid excision and conjunctival map biopsy identified DM of the eyelid. The risk of developing melanoma in NF1 is elevated, with both cutaneous and ocular variants of melanoma being observed in individuals with this condition [43,44]. Additionally, in certain melanoma cells, genetic analysis indicates mutations in the 17q11.2 locus of the NF1 gene [45].

An important question raised during the longitudinal follow-up of this patient is whether the lesion represented a single evolving pathological entity or two distinct processes occurring sequentially. The initial biopsy demonstrated immunophenotypic features compatible with melanoma, including positivity for S100P and Melan-A, with focal HMB-45 expression. However, subsequent specimens consistently showed S100P and SOX10 positivity, while Melan-A and HMB-45 became negative, and CD34 expression was identified after surgical excision.

Compared with previously reported cases of desmoplastic melanoma and neurofibroma, our patient exhibited similar immunohistochemical features, including S100 and SOX10 positivity, as described in Yeh et al. and Rubinstein et al. [42,43]. However, our case differed in the longitudinal evolution of marker expression, with initial Melan-A positivity transitioning to negativity over time, and persistent CD34 expression characteristic of NF. This contrasts with typical DM cases, where CD34 expression is generally absent or patchy. Moreover, the protracted diagnostic trajectory in our patient, involving multiple biopsies and surgical procedures over seven years, highlights how diagnostic challenges may be amplified by overlapping histopathological features and evolving immunophenotypes, whereas other reported cases often achieved definitive diagnosis more rapidly.

This evolving immunoprofile raises the possibility of either diagnostic overlap between desmoplastic melanoma and neurofibroma or biological modification of the lesion over time. It is well recognized that desmoplastic melanoma may lose expression of melanocytic markers such as Melan-A and HMB-45 in recurrent or progressive lesions, while maintaining S100 and SOX10 positivity. Conversely, neurofibromas consistently demonstrate S100 and SOX10 expression and may show CD34 positivity.

Therefore, despite initial postoperative regression observed in certain MRI planes, the presence of margin involvement and imaging variability highlights the potential for residual or progressive disease. Long-term clinical and radiological follow-up is mandatory in such cases to monitor for recurrence or further tumor progression.

### Limitations

Moreover, immunohistochemical expression patterns are not always static. Tumor evolution, treatment effects (including surgical manipulation and radiotherapy), sampling variability, and clonal selection may contribute to changes in marker expression over time. Therefore, variability in immunophenotype does not necessarily confirm recurrence of the identical pathology but may reflect biological progression, phenotypic modulation, or the coexistence of overlapping entities.

In our case, the longitudinal immunohistochemical findings, together with the clinical course, suggest a complex diagnostic trajectory rather than a straightforward recurrence of a single stable pathology.

## 5. Conclusions

In summary, the similarities in clinical presentation, histopathological features, and shared risk factors between melanoma and NF1 contribute to the difficulty in differentiating these two conditions. A multidisciplinary approach involving dermatologists, oncologists, and pathologists may be necessary to accurately diagnose and manage patients with suspected melanoma or NF1.

## Figures and Tables

**Table 1:** Biopsy results.

Biopsy No.	Date	Description of Microscopic Examination:	Conclusion
1	13 April 2017	The resulting small fragments are covered with the epidermis, where the pagetoid spread of tumor cells is observed. Nested tumor cells with round nuclei with an eosinophilic nucleus, an uneven nuclear contour, and moderately abundant eosinophilic cytoplasm are seen in the area of the junction of one piece. Tumor cells are positive for S100P and Melan-A immunolabels; some cells react with HMB45 immunolabel. Tumor cells do not react with CK5, CD1a, and CD68 immunolabels.	Suspicion of malignant melanoma.
2	29 June 2017	A fragment of tissue covered by the epidermis transitioning to the conjunctiva. Lentiginous proliferation of atypical epithelioid melanocytes, intraepidermal spread of individual cells, and invasion in the dermis are visible in the area of the epidermal junction. Elongated and spindle-shaped cells are visible in the dermis, arranged in fibers—could be fibroblasts or desmoplastic melanoma. Tumor cells react with the S100 marker. Part, in the junctional area and several sockets in the dermis are positive for HMB-45. Melan-A does not react with smooth muscle actin, CD34, EMA, CD56, Synapt, and CD68, Ki 67—proliferative index is high in the junction area, lower in the dermis, assessment is complicated by abundant lymphocytic infiltration. Tumor thickness according to Breslow—at least 1.05. The radicality of removal of epithelioid cells is questionable.	Malignant melanoma of the skin pT2a (at least).
3	10 June 2019	The obtained fragment is covered with stratified squamous epithelium. In the dermis/stroma, small nerve fibers and concentric groups of fibers around them and single spindle-shaped cells with oval nuclei and moderately abundant cytoplasm are visible. Focal lymphocytic infiltration is visible under the epithelium, in the dermis. Spindle cells and small nerve fibers are positive for the S100P immunolabel. Spindle-shaped cells do not react with EMA, CK markers. LCA marker highlighted inflammatory elements. Proliferation index Ki67 up to 2%.	Benign lesions, most data for traumatic neuroma.
4	17 March 2022	The resulting fragment shows small nerve fibers in the dermis/stroma and concentrically arranged groups of fibers and single spindle-shaped cells with oval nuclei, moderate abundance of cytoplasm. Spindle-shaped cells and small nerve fibers are positive for S100P immunolabel. The reaction with total CK, HMB45, and Melan-A markers is negative. Proliferation index in Ki67 spindle-shaped cells < 2%.	Benign changes, mostly data for traumatic neuroma.
5	21 September 2022	In the obtained soft tissue fragment covered with the epidermis, a fairly well-demarcated, medium-cellular tumor formation without a clear capsule was found, formed by irregularly arranged, mitotically low-active cells with plump oval nuclei and an average amount of oxyphilic cytoplasm with unclear boundaries. The majority of tumor cells react positively with immunolabels S100 and SOX10. About 30 percent cells observed weakly positive nuclear reaction with LEF1. Thirty percent cell-positive P16. The immunoproliferative marker is positive in about 20 percent cells. Some of the smaller cells with an elongated nucleus react positively with EMA. Reactions with Desmin, Melan-A, ALK, H3, UCHL-1,5, NSE2, HMB-45, GFAP, Beta-Catenin, Actin, Synaptofsin are negative. No activating BRAF V600 mutation was found in the tumor cells (the study was performed with the “Easy PGX” apparatus, using the real-time PCR method). In the tumor, scanty infiltration with monomorphonuclear cells was observed. Tumor cells surround sparse mature desmin-positive striated muscle cells present in the tumor.	Benign changes, mostly data for traumatic neuroma.
6	10 February 2023	In the columns of the tissue, small spindle-shaped cells with oval (minimally polymorphic) nuclei and cytoplasm of moderate abundance are visible. Spindle-shaped cells positive for S100P, SOX10 immunolabels. EMA-positive fibers are seen in places around the spindle-shaped cells. Single nerve fibers are found with the PGP9.5 marker. Immunohistochemical reactions with general CK, actin, PR, Melan-A, HMB45 markers are negative. Proliferation index Ki67 up to 2–3%.	Most data for benign hybrid nerve sheath tumor.
7	26 April 2023	A relatively well-circumscribed, well-vascularized tumor was obtained with small fibrous, mitotically inactive, spindle-shaped cells with oval (minimally polymorphic) nuclei and moderately abundant cytoplasm. The majority of cells are positive for the SOX10 immunolabel. Proliferation index Ki67 up to 5%.	Benign changes, possibly neurofibroma (possible component of a hybrid tumor of nerve sheaths).
8	29 October 2024	The obtained material revealed a cellular—moderately cellular tumor that overgrows the eyeball, spreads to the eyelids, infiltrates the muscle tissue, and in some places reaches the color-marked edges of the fragment, formed by fibers, in some places of storiformly arranged elongated or polygonal tumor cells with a large oval / elongated nucleus and a moderate amount of cytoplasm. Mitoses are rare. Moderately expressed infiltration with monomorphonuclear cells is observed in the stroma. Tumor cells react positively with immunomarkers S100, p16; reactions with EMA, calretinin, CD34 are negative. Ki-67 activity is low, 5–10 percent.	Left orbital tumor: Benign tumor of the peripheral nerve sheath—most data for neurofibroma.

**Table 2:** Description of magnetic resonance imaging (MRI) in the past two years.

Magnetic Resonance Imaging No. 1	
Date:	17 August 2022
	On the left extraconal medial additional formation, intensively accumulating contrast agent (suspected melanoma, although the referral states that it was suspected and denied); intracranially without visible pathological MR SI changes; hypoplastic right maxillary fissure, moderately thickened mucosa in both maxillary fissures.
Magnetic resonance imaging No. 2	
Date:	21 December 2022
	On the left, an extraconal lateral, pre- and post-septal intensive contrast agent accumulating formation (accurate histological verification), possibly infiltrating the lower surface of the lacrimal gland.
Magnetic resonance imaging No. 3	
Date:	7 June 2023
	Condition under left orbital tumor excision 26 April 2023, in the postoperative area, according to MR, a recurrence is suspected against the background of postoperative, edematous changes—purposeful histological verification. On the right side of the eyelids, the infiltrative changes at the slit are non-specific, without essential MR dynamics, to be decided on the verification of the possible process.
Magnetic resonance imaging No. 4	
Date:	27 May 2024
	Progression of tumor multifocal infiltration on the left intra-extraorbital. Similar to pathologic preauricular lymph node on the left—to be examined morphologically.
Magnetic resonance imaging No. 5	
Date:	14 August 2024
	Signs of progression of the oncological process.
Magnetic resonance imaging No. 6	
Date:	8 January 2025
	In the left temporal fossa—perizygomatically and in the left parotid gland area, the volume of tumor formations has decreased; in the suprazygomatic area of the temporal fossa, individual formations should be differentiated between new foci or fragmentation caused by a previous formation.

## Data Availability

The original contributions presented in this study are included in the article. Further inquiries can be directed to the corresponding author.
